# Environmental Potential of Carbonized MOF-5/PANI Composites for Pesticide, Dye, and Metal Cations—Can They Actually Retain Them All?

**DOI:** 10.3390/polym15224349

**Published:** 2023-11-07

**Authors:** Anka Jevremović, Marjetka Savić, Aleksandra Janošević Ležaić, Jugoslav Krstić, Nemanja Gavrilov, Danica Bajuk-Bogdanović, Maja Milojević-Rakić, Gordana Ćirić-Marjanović

**Affiliations:** 1 Faculty of Physical Chemistry, University of Belgrade, Studentski trg 12-16, 11158 Belgrade, Serbia; 2 Vinča Institute of Nuclear Science, National Institute of the Republic of Serbia, University of Belgrade, P.O. Box 522, 11001 Belgrade, Serbia; 3 Faculty of Pharmacy, University of Belgrade, Vojvode Stepe 450, 11221 Belgrade, Serbia; 4 Department of Catalysis and Chemical Engineering, Institute of Chemistry, Technology and Metallurgy, University of Belgrade, Njegoševa 12, 11000 Belgrade, Serbia

**Keywords:** polyaniline, MOF-5, cadmium, composite, Methylene Blue, acetamiprid, adsorption, N,O-doped carbon, ZnO

## Abstract

The environmental application of the carbonized composites of the Zn-containing metal-organic framework MOF-5 and polyaniline (PANI) in its emeraldine salt and base forms (C-(MOF-5/PANI)) was investigated for the first time. Textural properties and particle size distributions revealed that composites are dominantly mesoporous and nanoscale in nature, while Raman spectroscopy revealed the ZnO phase beneath the carbon matrix. Adsorption of pesticide, dye, and metal cation on C-(MOF-5/PANI) composites in aqueous solutions was evaluated and compared with the behavior of the precursor components, carbonized MOF-5 (cMOF), and carbonized PANIs. A lower MOF-5 content in the precursor, a higher specific surface area, and the pore volume of the composites led to improved adsorption performance for acetamiprid (124 mg/g) and Methylene Blue (135 mg/g). The presence of O/N functional groups in composites is essential for the adsorption of nitrogen-rich pollutants through hydrogen bonding with an estimated monolayer capacity twice as high as that of cMOF. The proton exchange accompanying Cd^2+^ retention was associated with the Zn/Cd ion exchange, and the highest capacity (9.8 mg/g) was observed for the composite synthesized from the precursor with a high MOF-5 content. The multifunctionality of composites was evidenced in mixtures of pollutants where noticeably better performance for Cd^2+^ removal was found for the composite compared to cMOF. Competitive binding between three pollutants favored the adsorption of pesticide and dye, thereby hindering to some extent the ion exchange necessary for the removal of metal cations. The results emphasize the importance of the PANI form and MOF-5/PANI weight ratio in precursors for the development of surface, porosity, and active sites in C-(MOF-5/PANI) composites, thus guiding their environmental efficiency. The study also demonstrated that C-(MOF-5/PANI) composites retained studied pollutants much better than carbonized precursor PANIs and showed comparable or better adsorption ability than cMOF.

## 1. Introduction

Environmental studies nowadays extend their area not only to monitor and limit the hazardous influence of anthropogenic actions but also to establish a neat connection to advanced material science needed for tackling these issues. The remediation process must include the retention and safe disposal of contaminated materials. Although solid crystalline products are considered broadly applicable in remediation procedures, particularly contaminated waters, novel routes and different approaches in the material science field are essential for environmental solutions [1,2,3,4]. In a search for tunable, well-defined structures, metal-organic frameworks (MOFs) present a class of highly potent adsorbents/catalysts for water pollutant removal [5,6]. Their applicability in environmental studies recently emerged in the removal of different pollutants: dyes [7,8], toxic metals [9,10], and pesticides [11], among others [12]. Is it possible to introduce such functionalities during synthesis and/or post-synthetic modification to produce materials able to accommodate different pollutant structures? For instance, the Cd^2+^ coordination with the electron donor is expected, thus pointing to the beneficial introduction of nitrogen to the MOF structure [13]. Fang et al. proposed MOF synthesized with amino-containing ligands for Cd removal [14]. Amino-functionalization was also proved to be useful for MOF material—UiO (Universitetet i Oslo) [15] tested for the adsorption of different toxic metals. Dithizone immobilized MOF was applied for solid-phase extraction of Pb and Cd in wastewater samples [16]. Furthermore, components that increase dispersibility in an aqueous environment and introduce novel active sites for adsorption usually comprise nitrogen-containing functional groups, often witnessed for dye removal [8,17,18].

Since amines readily adsorb pollutants, boosting the pristine MOF potential for removal may be a promising line of action. Namely, introducing highly functional components in the MOF structure, such as polyaniline (PANI), a nitrogen-rich conductive polymer, has made some significant contributions to state-of-the-art materials [19,20,21,22,23]. Favorable charge distribution around nitrogen will assist pollutants’ accommodation, possibly via hydrogen bonding, and enhance their removal [24,25]. Complex MOF structures can be sensitive to humidity and pH adjusting to different environments may affect their stability. To enhance MOF stability and introduce a range of functionalities, a combination of promising precursors is explored [26,27]. Recently, a series of MOF-5/PANI composites showed interesting features, such as remarkable surface area, conductivity, and high microporosity [28]. Moreover, the carbonization of such structures enabled the formation of electroactive N,O-doped carbon/ZnO-type composite materials, C-(MOF-5/PANI), with preserved high surface area, where PANI was the source of N heteroatoms [29]. In this way, multifunctional carbons doped with two heteroatoms (N, O) were obtained, which enables them to be widely used in environmental studies [30] targeting both efficient pollutant removal and application in energy storage [29].

The MOF carbonization procedure enables the formation of not only metal particles but also metal oxides surrounded by a carbon structure [31]. Given that these structures are considered non-toxic, they may find application in pollutant removal from water. First of all, the model substances that are tested in these conditions start from dyes [31], although their presence in the environment may not be as abundant as one seen for toxic metals and pesticides. To investigate further applicability of these specific, multifunctional structures, in this study, we have tested the removal of three classes of the most abundant pollutants in the aquatic environment—toxic metal (Cd^2+^), cationic dye (Methylene Blue), and pesticide (acetamiprid) by C-(MOF-5/PANI) composites, derived by the carbonization of composites prepared from MOF-5 and PANI in its emeraldine salt (ES) and base (EB) forms, which represent N,O-doped carbon/ZnO/ZnS and N,O-doped carbon/ZnO composites, respectively. Testing of carbonized precursor components MOF-5 and PANIs under the same conditions was also conducted for comparison.

## 2. Materials and Methods

### 2.1. Materials Syntheses

The syntheses of MOF-5, PANI, and MOF-5/PANI composites are performed according to procedures reported in refs. [28,32], with p.a. chemicals supplied by Centrohem (Stara Pazova, Serbia). In short, zinc acetate was employed for the preparation of MOF-5, using terephthalic acid (benzene 1,4-dicarboxylic acid) as a linker, N,N’-dimethylformamide (DMF) as a solvent, and chloroform for activation of produced MOF-5 by solvent exchange [32]. Two forms of PANI, synthesized by the oxidative polymerization of aniline with ammonium peroxydisulfate (APS), were used for MOF-5/PANI composites preparation: conducting emeraldine salt, ES, synthesized in the solution of HCl (0.2 M aniline hydrochloride, 0.25 M APS) and nonconducting emeraldine base, EB, synthesized in water without added acid (0.2 M aniline, 0.25 M APS) and dedoped by an excess of alkali, according to refs. [28,29]. The composite is denoted as MOF.ESb, with 77 wt.% MOF-5 content, was prepared by mechanical mixing of MOF-5 and ES components in the presence of chloroform [28]. Composites containing EB were prepared by mixing the dissolved part of EB in DMF with MOF-5; they contained 71, 77, and 89 wt.% MOF-5 and are denoted as MOF.EBa, MOF.EBb, and MOF.EBc, respectively. Prepared MOF-5/PANI composites and their starting components, MOF-5, ES, and EB, are carbonized using a tube furnace (CP-45, Elektron, Serbia), by gradual heating in an argon atmosphere with a heating rate of 10 °C/min up to 800 °C. The samples obtained by carbonization are denoted by using the prefix *c* in the names of corresponding precursors, i.e., cMOF, cES, cEB, cMOF.ESb, cMOF.EBa, cMOF.Ebb, and cMOF.EBc.

### 2.2. Materials Characterization

Nanoparticle Tracking Analyzer (NTA) NanoSight 300 (Malvern Panalytical, Malvern, UK) was employed for particle size determination in the sample suspension using a dynamic mode. For optimal results, 5 captures over 60 s time intervals were recorded for each sample and statistically treated to obtain mean particle size. All samples were suspended in water to acquire 2–8 × 10^8^ particles/mL concentration and were sonicated before measurement. After capture, the videos were analyzed by the inbuilt NanoSight NTA 3.4. software.

Raman spectra and microanalytic spectral maps of the studied solid samples were recorded on a DXR Raman spectrometer (Thermo Scientific, Waltham, MA, USA) comprising an optical microscope and a CCD detector, using laser excitation at a wavelength of 532 nm. Laser power was set to 1 mW, at exposure of 10 s in 60 repetitions. When examining the effect of laser power on the spectra, selected power outputs were 2, 5, and 10 mW, with 3 × 5 s scans. The mapping was performed with a laser power of 10 mW, a map size of 90 μm × 90 μm, and a step size of 10 μm, resulting in 100 recorded spectra. An 800 lines/mm grating and 50 μm pinhole aperture were selected for all measurements. Deconvolution of Raman spectra was performed in Omnic 9 software (Thermo Scientific, Waltham, MA, USA). 

The Sorptomatic 1990 Thermo Finnigan sorption analyzer (Thermo Scientific, Waltham, MA, USA) was used to record the nitrogen adsorption-desorption isotherms for the tested materials. The obtained isotherms were analyzed using the ADP 6.0 Thermo Electron software package. To determine the total pore volume, the Gurvich method was applied, specifically considering a relative pressure (p/p^0^) of 0.98. For the analysis of mesopores, the desorption isotherm curve was analyzed using the Barrett-Joyner-Halenda (BJH) method [33]. The microporosity was investigated according to the Horvath and Kawazoe method [34]. Estimated errors of the measured textural parameters using the Sorptomatic 1990 instrument do not exceed 3% [35].

### 2.3. Adsorption Study

Each pollutant concentration for the batch adsorption test was selected to enable differentiation of the adsorption performance of tested materials.

*Acetamiprid pesticide.* The suspension for the batch adsorption test comprised 2 mL of 200 mg/L acetamiprid solution (99%, AgroSava Ltd., Belgrade, Serbia) with a 1:1 solid-to-liquid ratio. Equilibration time was 24 h for suspensions stored on a laboratory shaker at 110 rpm and 23 °C. After centrifugation and filtration through 0.45 µm nylon filters, the remaining pesticide was quantified using the HPLC system (Bischoff, Leonberg, Germany) equipped with Compact Pump 2250, LC-CaDI 22–14 Interface, injector, and Gastorr TG-14 Degasser. An isocratic eluent mixture of acetonitrile-water (50/50 *v*/*v*) with 1 mL/min flow and ProntoSIL 120-5 C18 AQ plus (Bischoff) column was used, while a Lambda 1010 detector was set at a wavelength of 245 nm for acetamiprid detection. Adsorption isotherms were recorded in low suspension volumes (2 mL), investigating a range of acetamiprid concentrations up to 200 mg/L. 

*Methylene Blue dye.* The batch dye adsorption test included 2 mg of tested materials and 2 mL volume of 200 mg/L Methylene Blue (MB) (Merck, Rahway, NJ, USA) solution. The suspensions were placed on a laboratory shaker (OLS Shaking bath, Grant Instruments) at 110 rpm and 23 °C for 24 h, afterward centrifuged at 13,400 rpm (Minispin Eppendorf, Hamburg, Germany), and UV-Vis spectra were recorded on Evolution 220 (Thermo Scientific, Waltham, MA, USA).

*Cadmium ions.* The adsorption capacities of the studied materials were evaluated for Cd ions in a batch adsorption system with pH optimization. Acidity adjustment was performed with standard 0.1 M NaOH/HCl solutions. Along with literature data, suspensions were placed in glass vials containing 5 mL of 10 mg/L Cd^2+^ concentration (3CdSO_4_⋅8H_2_O, Thomas Tyrer & Co., Stirling, Scotland), guided by its environmental occurrence [36], and 5 mg of each sample. The adsorption experiments were performed at constant temperature in a continuous stirring mode at 110 rpm on the laboratory shaker (OLS200, Grant Instruments) for 24 h. Afterward, suspensions were centrifuged at 13,400 rpm, and the Cd^2+^ amount was quantified, in the supernatants, by atomic absorption spectrometry in flame operational mode at AAnalyst 700 (Perkin-Elmer, Shelton, CT, USA). Quantitative adsorption data are given as percent of initial metal content, calculated from the difference between initial and equilibrium Cd^2+^ quantity.

*Pollutant mixtures*—To test competitive binding, a selection of samples was tested as adsorbents in two-component (AA/MB and AA/Cd) and three-component (AA/MB/Cd) suspensions of pollutants. Samples loading was 1 mg/mL, with each component’s final concentration being set to 10 mg/L.

*Isotherm models*—Typical adsorption isotherm models are explored: empirical Freundlich (ascribed to heterogeneous surfaces and best fitted to low concentration isotherm end), theoretical Langmuir model (suited for energetically uniform surfaces that allow ideal, monolayer coverage of adsorbent) and the combination thereof, Langmuir–Freundlich model, q_e_ = Kq_m_C_eq_^1/n^/(1 + C_eq_^1/n^), reported to best describe composites and functionalized carbons applied in environmental studies [24]. The equilibrium adsorbate concentration is measured after adsorption C_eq_ (mg/L), while q_e_ (mg/g) is the amount of adsorbate (in mg) retained by a gram of adsorbent at a given C_eq_; q_m_ is the maximum amount of adsorbate estimated for the monolayer (mg/g), K (L^n^/mg^n^) is the adsorption constant, and 1/n is used as a surface heterogeneity estimate, approaching 1 for uniform surfaces as Langmuir–Freundlich transfers to Langmuir model. The nonlinear fit of adsorption isotherm data was performed in the OriginPro2021 program, while the fitting quality was assessed using the coefficient of determination, R^2^.

## 3. Results and Discussion

### 3.1. Particle Size

**Figure 1:**
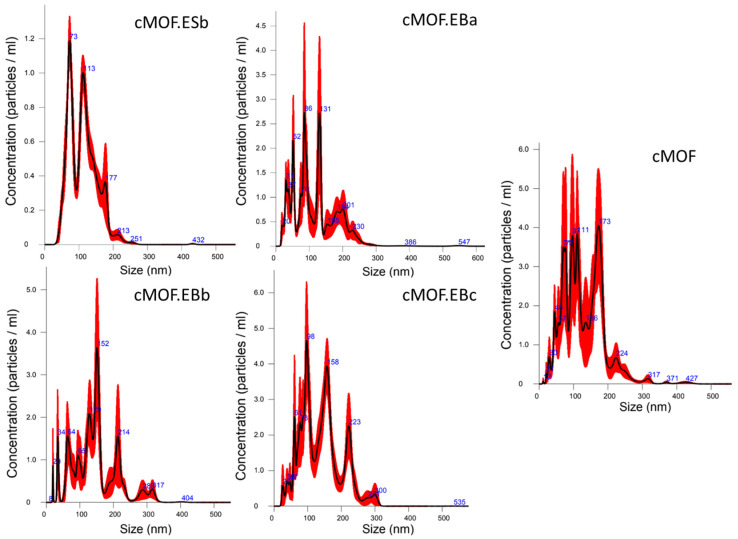
Averaged FTLA Concentration/Size curves, determined by NTA, for tested C-(MOF-5/PANI) composites and cMOF sample. Error bars indicate +/− standard error of the mean.

For the analysis of the adsorption performance and the understanding of the remediation process, the data on particle size and the way the particles behave in the solution/dispersion are of great importance. NTA is a technique suitable for the analysis of nanoparticles in liquid suspensions, providing the particle size distribution of samples with high resolution. The particular advantage of this technique compared to static analysis is reflected in the visual tracking of individual particles in motion, allowing changes such as particle aggregation to be monitored [37,38]. Particle size distributions of the studied samples determined by NTA revealed that C-(MOF-5/PANI) composites and cMOF are nanomaterials whose mean particle sizes are in the range of 112–145 nm. Averaged Finite Track Length Analysis (FTLA) of particle concentration vs. size was recorded, Figure 1. 

The smallest mean particle size is measured for the composite derived from an ES-containing precursor, cMOF.ESb (112 nm), and the composite derived from the EB-containing precursor with the lowest MOF-5 content, cMOF.EBa (116 nm). 

Starting from the cMOF.ESb sample, with a narrow particle size distribution, the curves widen for the composites of the cMOF.EB sample series, Figure 1. The cMOF.EBb and cMOF.EBc samples show mean particle sizes of 145 and 139 nm, respectively, while a similar value is obtained for the cMOF sample, 132 nm. These differences in the mean particle size and particle size distribution of the tested materials may be associated with the more hydrophilic surface of cMOF.ESb, indicated by thermogravimetric measurements [29], and its better dispersibility in water, in comparison to the cMOF.EB samples. 

### 3.2. Textural Properties

The textural properties of the investigated materials reflect the initial composite compositions. Previously, it was reported that tested composites have a high portion of cuboid particles, where the high MOF-5 content in the composite precursor (>70 wt.%) enabled the development of a large surface in C-(MOF-5/PANI) produced by carbonization [29]. The S_BET_ values were 609, 470, and 412 m^2^/g for cMOF.EBa, cMOF.EBb and cMOF.EBc, respectively, and 393 m^2^/g for cMOF.ESb [29]. For the carbonized individual precursor components, S_BET_ amounted to 553 m^2^/g (cMOF), 351 m^2^/g (cES), and 273 m^2^/g (cEB) [29]. We determined the additional textural properties relevant to the adsorption behavior: total pore volume (V_tot_), micropore volume (V_mic_), mesopore volume (V_meso_), and median diameter (D_meso_) are summarized in Table 1. Adsorption/desorption isotherms, with the accompanying pore size distribution curves for mesopores (B. J. H. model), are depicted in Figures S1 and S2. The highest V_tot_ (1.106 cm^3^/g) is found for cMOF.EBa and decreases with increasing MOF-5 content in the MOF.EB precursor composite (i.e., with increasing ZnO content and decreasing carbon content in cMOF.EB) to 0.359 cm^3^/g for cMOF.EBc. The lowest value of V_tot_ is recorded for cMOF.ESb (0.315 cm^3^/g). For all C-(MOF-5/PANI) samples, mesoporosity is prevalent over microporosity with the mesopore diameter rising with lowering MOF content in the precursors of the cMOF.EB sample series. The mesopores vs. micropores volume ratio, V_meso_/V_mic_, is the highest for the cMOF.EBa sample (c.a. 4.2), while other composites have comparable V_meso_ and V_mic_. Interestingly, cMOF has noticeably lower V_tot_, V_meso_, D_meso_, and V_meso_/V_mic_ ratio (c.a. 2.6) than the composite cMOF.EBa, implying that the introduction of PANI into the precursor of carbonization, in the appropriate amount and form, led to an increase in the overall porosity and mesoporosity of the carbonization product, C-(MOF-5/PANI).

### 3.3. Raman Spectroscopy

**Figure 2:**
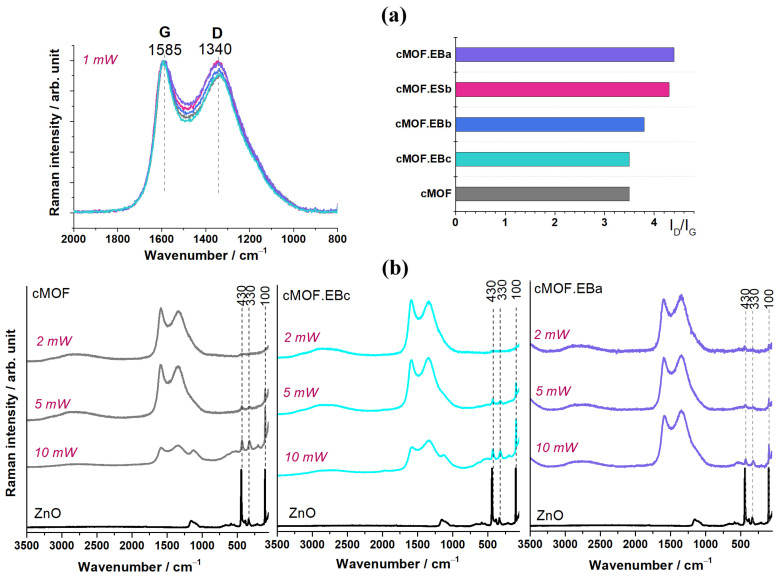
(**a**) Raman spectra with I_D_/I_G_ ratio graph of C-(MOF-5/PANI) composites and cMOF and (**b**) Raman spectra of cMOF, MOF.EBc, and cMOF.EBa samples collected at different laser powers (2, 5, and 10 mW), exhibiting the bands assigned to ZnO vibrations. The spectrum of pristine ZnO is also shown, recorded at 10 mW.

Raman spectroscopy can be used as a powerful tool to monitor the carbonization process of MOFs, PANIs, and their composites and to investigate the structure of obtained products [26,39]. The intensity ratio of the D and G Raman bands, I_D_/I_G_, can be used as a criterion for evaluating the content of defects on the surfaces of carbon materials. From Figure 2a, it can be seen that the G (graphitic) band in the Raman spectra of all the tested composites and cMOF is located at 1585 cm^−1^, while the D band, which is defect-dependent, is positioned at 1340 cm^−1^ [40]. Differences in the I_D_/I_G_ values indicate differences in the number of defects/disorder-induced structures produced during the carbonization of MOF-5 and its composites with PANI. The calculated values of the I_D_/I_G_ ratio for cMOF.EBa (4.4), cMOF.ESb (4.3), and cMOF.EBb (3.8) are higher than those of cMOF (3.5) and cMOF.EBc (3.5), Figure 2a. The obtained values for the composites are close to the previously reported I_D_/I_G_ values determined under different experimental conditions [29]. The calculated values above three were often reported for carbons derived from PANI salts [41] and revealed notable disorder, mainly attributed to the inclusion of N and O heteroatoms within the carbon network. Similar types of defects are expected in the carbonized composites tested here, due to PANI-originating N-containing functionalities (since cMOF does not contain nitrogen) and O-containing functionalities, originating from both PANI and MOF-5. Accordingly, a higher I_D_/I_G_ ratio was calculated for composites cMOF.EBa, cMOF.ESb, and cMOF.EBb (produced from the precursors with lower MOF-5 content, 71 and 77 wt.%) compared to I_D_/I_G_ for cMOF can be explained mainly by the influence of the PANI component, which introduces additional structural disorder in composites via N-containing species. The influence of PANI on I_D_/I_G_ is negligible in the case of cMOF.EBc produced with the highest amount of MOF-5 component (89 wt.%)

The Raman spectra of carbons are often susceptible to laser-induced alterations. The influence of increasing laser power on the Raman spectra of the cMOF, cMOF.EBc, and cMOF.EBa samples is shown in Figure 2b. It can be seen that at a low laser power of 2 mW, the G and D bands are neatly resolved in the spectrum. With increasing laser power to 5 mW, the intensity of the D band increases related to the G band, and, simultaneously, the bands of ZnO(at 430, 330, and c.a. 100 cm^−1^) become distinct, Figure 2b. At a laser power of 10 mW, the mentioned ZnO bands become even stronger (related to the G and D bands), and three additional bands of ZnO appear at 1123, 566, and 198 cm^−1^. Since a fraction of the sample volume is investigated by this surface analysis method, by removing the carbonaceous surface layers using high laser power, the bands of the underlying ZnO phase can be clearly distinguished. The mentioned bands attributed to ZnO fit well those observed in the spectrum of pure ZnO (Figure 2b). In the ZnO spectrum, the two intense, sharp bands assigned to E_2_ modes can be observed at 100 cm^−1^ (E_2low_) and 430 cm^−1^ (E_2high_), [42]. The band at 330 cm^−1^ corresponds to the second-order scattering, the E_2high_−E_2low_ mode, confirming the temperature dependence of Raman intensity [42]. The broad band at c.a. 1123 cm^−1^ was attributed to 2LO phonons, while the bands at c.a. 566 and 198 cm^−1^ were assigned to the A1(LO) and 2E_2low_ modes, respectively [43].

**Figure 3:**
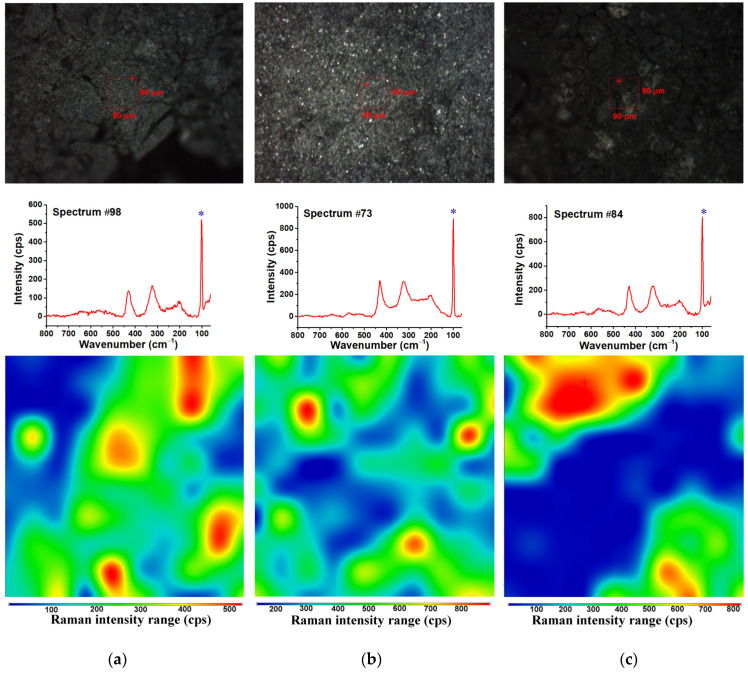
The micrographs of (**a**) cMOF, (**b**) cMOF.EBc, and (**c**) cMOF.EBa samples; the red rectangle corresponds to the mapped surface (**top**); the Raman spectrum obtained at the position marked with + (**middle**) and the Raman map with the intensity distribution of the band at 100 cm^−1^ (**bottom**), marked with * in the middle spectrum.

Raman maps of the cMOF, cMOF.EBc, and cMOF.EBa samples are shown in Figure 3. The intensity distribution for the band at 100 cm^−1^ (marked with * in the middle spectra) is shown in Figure 3 bottom, implying a more homogeneous dispersion of the ZnO phase in the cMOF and cMOF.EBc samples with high MOF content in the precursor before carbonization. On the other hand, in the cMOF.EBa sample, derived from the composite with lower MOF content, ZnO has less homogeneous occurrence beneath the carbonaceous layer.

### 3.4. Pesticide Adsorption

Adsorption studies often involve independent testing of the optimal starting concentration, reaction time, and adsorbent dose without critical insight into the adsorption phenomenon. Lowering of adsorbate concentration and adsorbent amount will boost the apparent adsorption performance. That is why the adsorbent dose testing can be comprehensively covered by adsorption isotherm modeling with a reasonable solid-to-liquid ratio (in the proximity of several mg/mL). Pollutant concentration in batch adsorption tests needs to be sufficiently high to discriminate among different procedures for materials preparation/optimization. Then the adsorption isotherm modeling gives an insight into adsorption sites’ homogeneity. After this starting step, the experiment should be conducted in a low concentration range to address the real amount of contaminants present in the environment.

**Figure 4:**
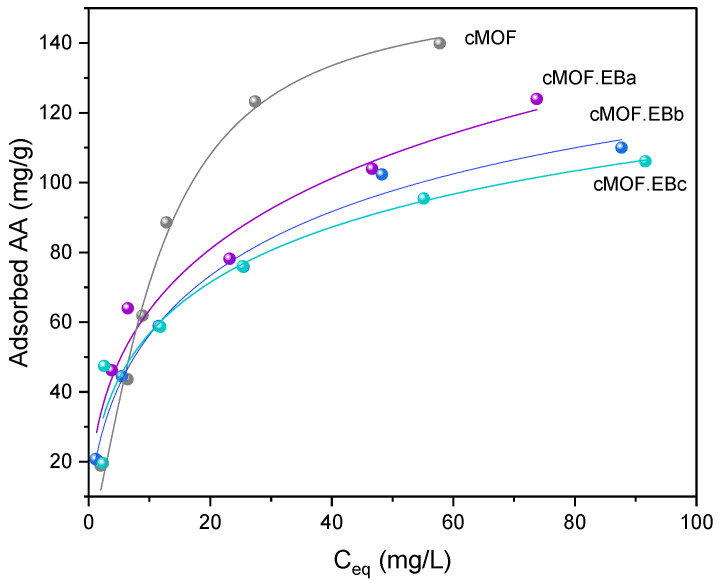
The results of AA adsorption on cMOF.EB composites and cMOF, fitted with the Langmuir–Freundlich isotherm model.

The investigation of the applicability potential for studied materials starts with a pesticide, an emerging environmental contaminant. As a representative, a neonicotinoid insecticide, acetamiprid (AA), was used. The fitting results for AA adsorption isotherms for cMOF.EB samples and cMOF are shown in Figure 4. A good correlation is established with the applied Langmuir–Freundlich model, 0.93–0.99. The estimated monolayer retention is the highest for cMOF.EBa (298 mg/g), followed by cMOF.EBc (183 mg/g) and cMOF.EBb (181 mg/g). For the cMOF sample, saturation is almost reached at experimental values, with monolayer retention achieved at 155 mg/g. 

All tested composites and cMOF have proved to be excellent for AA adsorption. Composites of the cMOF.EB series have reached the adsorption capacity of over 100 mg/g, which is superior to the cMOF.ESb sample capacity (71 mg/g). Interestingly, lower MOF-5 content in the composite precursor (i.e., lower ZnO content in carbonized composite) leads to better adsorption capacity of cMOF.EB composite (Figure 4), while cMOF has the highest capacity (140 mg/g), closest to the value recorded for the composite synthesized with the lowest MOF-5 content, cMOF.EBa (124 mg/g). The reason for the highest capacity of cMOF.EBa among all composites may be found in its highest specific surface area S_BET_ and highest pore volumes (V_tot_, V_meso_, and V_mic_), Table 1. The other two composites, cMOF.EBb and cMOF.EBc, have lower S_BET_ and pore volumes, with especially lower V_meso_ (Table 1), which support the dominant role of the developed surface area and mesoporosity for pesticide adsorption. However, the surface area is not the only feature we need to look into. Sample cMOF.ESb developed a rather high S_BET_, nearly 400 m^2^/g, but showed significantly lower AA retention. Since we have shown that pristine cES does not adsorb AA at all, it implies that the crucial part of the cMOF.ESb composite responsible for the AA adsorption is a carbonized MOF-5 component of the precursor. On the other hand, cEB showed a capacity of 39 mg/g, thus participating in the retention of AA along with the carbonized MOF-5 component of the composite, thus leading to higher capacities of cMOF.EB composites. This confirms the importance of the PANI form in precursors for the development of surface, pores, and adsorption sites in derived functional carbons. Although neat cMOF has proved to be the best adsorbent for AA at high pesticide concentrations (200 mg/L), the composite cMOF.EBa has significant retention abilities and matching results at environmentally significant AA loadings (Section 3.7). Moreover, the estimated isotherm monolayer capacity of cMOF.EBa composite is twice the value calculated for cMOF, pointing to its excellent performance. 

Envisaged adsorption sites for the removal of AA are dominantly oxygen and nitrogen-containing groups at the surface (e.g., C-N and O-H [29]). The calculated surface at.% ratio of oxygen/nitrogen is 9.6/7.0 (cMOF.EBa), 11.0/3.0 (cMOF.EBb), 8.3/2.5 (cMOF.EBc), and 6.7/8.2 (cMOF.ESb) from the EDX results [29]. Acetamiprid favors hydrogen bonding as the dominant adsorption mechanism [2], and this may also be a dominant interaction in this system due to N-containing (pyridinic, pyrrolic, quaternary, or tetrahedral N) and O-containing functionalities (C=O, OH, C-O-C, phenoxazine) in C-(MOF-5/PANI) composites, suitable for hydrogen bonding with AA via its N-containing groups (cyano and pyridinic). It is especially pronounced for cMOF.EBa with a high content of hydrogen bonding positions due to its highest S_BET_ and pore volumes as well as the highest summary surface content of oxygen and nitrogen. Hence, functional N,O-doped carbon/ZnO composites C-(MOF-5/PANI), rich in hydrogen bond forming groups, originating from MOF/PANI composites, may serve as a material of choice for AA removal. 

Different functional materials have been investigated so far for AA removal. When functional carbons are in question, biochar materials are promising for AA removal, reaching over 80 mg/g [44] with a low adsorbent dosage, similar to the one applied in this work. Higher adsorbent content may reduce removal efficiency for porous carbons to nearly 22 mg/g [45], while a significant amount of gram loading for clay minerals induces below 10 mg/g of this pesticide [46]. The significantly higher adsorption capacities of C-(MOF-5/PANI) composites point to their advanced surface features active for pesticide removal.

### 3.5. Dye Adsorption

**Figure 5:**
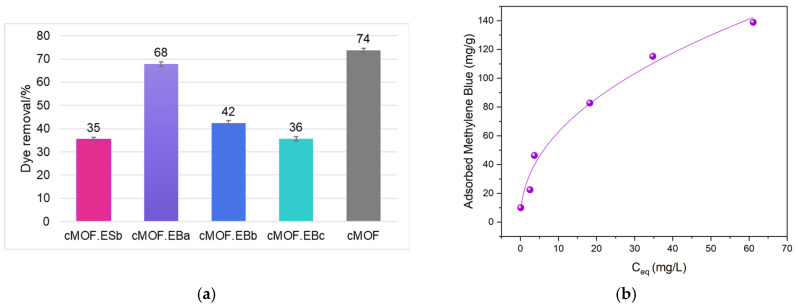
(**a**) The adsorption capacity of tested C-(MOF-5/PANI) composites and cMOF toward MB and (**b**) the adsorption isotherm for the cMOF.EBa sample.

Since tested C-(MOF-5/PANI) composites have proved to be high-functioning pesticide adsorbents, the investigation proceeded toward the dye contaminant. Methylene Blue (MB) was selected for the investigation of tested material adsorption capacity for colored, persistent pollutants. Carbonized precursor components show distinctly opposing behavior—cES showed 2.3 mg/g removal, cEB retained no dye, and cMOF outperformed both PANI-derived carbons (147 mg/g removal). The adsorption capacity of C-(MOF-5/PANI) composites and cMOF is summarized in Figure 5.

Comparable results are again seen for the cMOF.EBa (68% MB removal, i.e., 135 mg/g) and cMOF samples (74% MB removal, i.e., 147 mg/g), Figure 5a. With the MOF-5 content rising in the composite precursors of the cMOF.EB samples, adsorption is lowered to 42 and 36% MB removal for cMOF.EBb and cMOF.EBc, respectively. This trend is similar to the one seen for AA retention and is associated with a trend in surface and pore development. Interestingly, the role of the PANI precursor is reversed, and the comparable capacity of the cMOF.EBb and cMOF.ESb samples (with the same MOF content in the precursor composite) toward MB adsorption may be associated with minor, but detectable retention by the cES component, and similar S_BET_ values. Among the studied composites, the content of the carbonaceous part is the highest in cMOF.ESb [29], which enables π-π stacking with relatively planar MB molecules. An excellent correlation is witnessed with an applied Langmuir–Freundlich model, 0.99, confirming relative uniformity of adsorption sites accessible for MB dye retention, similar to one seen for AA and often recorded for functional carbons [47]. Since MB is a nitrogen-abundant compound, similar to AA, it can bind to the adsorbent with suitable adsorption sites through hydrogen bonding, as reported for materials of different surface chemistries [47,48,49]. Such adsorption sites in C-(MOF-5/PANI) composites are aforementioned various N-containing and O-containing functional groups [29]. It is reported that materials with small particle sizes and high surface areas may be suitable for the removal of MB [50]. Samples employed for these applications vary across the spectrum of conventional adsorbents, such as minerals [50], biopolymers [51], and biochars [52], reaching up to 130 mg/g. The materials tested here outperform various adsorbents and may be promising in water remediation. 

### 3.6. Metal Ions Adsorption 

The adsorption process is greatly affected by the pH of the solution due to its influence on the surface charge of the adsorbent and the redox potential vs. pH, i.e., speciation of the metal ions in the aqueous solution. For this reason, the acidity optimization for the adsorption test was performed for the selected samples (Supplementary Information, Figure S3).

**Figure 6:**
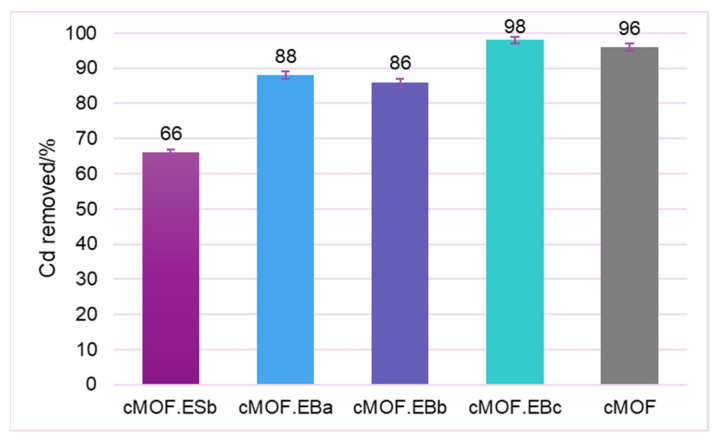
Percent of removed Cd after adsorption on C-(MOF-5/PANI) composites and cMOF at pH = 3.

The best adsorption capacity among the selected samples was observed in the cMOF.EBc sample for which almost complete Cd ions removal at pH 3 was achieved (98%, Figure 6) with somewhat lower adsorption at higher pH values of pH 5 and 8 (Figure S3). This finding, at first, appears contrary to the tendency observed in the literature for pristine ZnO [53]. However, adsorption found for investigated composites is associated with different protonation levels of the carbonaceous part and stability of the oxide phase located under the layer of carbonized PANI. Considering these results, the adsorption capacity evaluation of the studied materials proceeded at pH 3, Figure 6.

**Figure 7:**
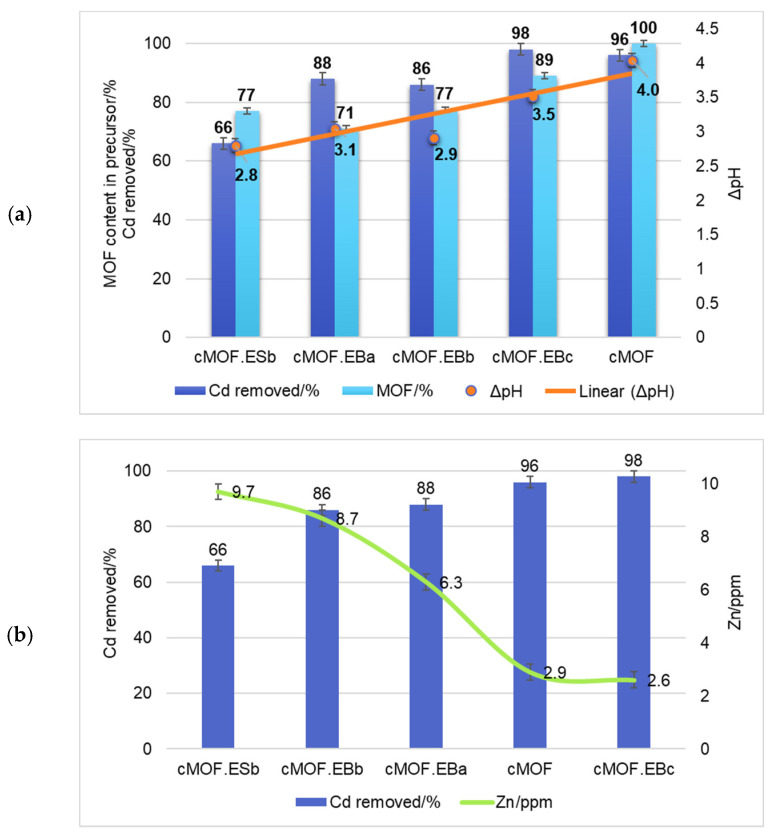
The efficiency of Cd^2+^ removal by C-(MOF-5/PANI) and cMOF samples, shown vs. (**a**) rising MOF-5 content in the MOF-5/PANI composite precursors before carbonization and pH change (ΔpH) during Cd^2+^ adsorption (with linear fitting of ΔpH) and (**b**) Zn^2+^ ions content in supernatants after Cd^2+^ ions adsorption.

The significantly lower performance of cMOF.ESb in comparison to the cMOF.EB samples appear to favor composites synthesized from MOF.EB precursors for the production of carbonized materials with specific textural properties that are efficient for Cd removal. To discriminate between the contribution from MOF-5 and PANI precursor to the adsorption capacity, we performed tests of Cd^2+^ removal on cEB and cES. Interestingly, both carbonized pristine PANI samples were inactive for Cd^2+^ removal, which suggests that composite precursor features offer the possibility for the development of efficient active sites after carbonization. The capacity of the samples for Cd^2+^ retention rises with MOF content in composites and the best result is recorded for cMOF.EBc (98% removal). This sample additionally offers the highest yield after carbonization [29] among tested samples, making it a viable choice for larger production. Excellent adsorption performance is also measured for cMOF (96% removal). To target active sites and mechanisms for Cd^2+^ retention, pH change during adsorption was monitored and shown versus Cd^2+^ removal efficiency and MOF-5 content in the precursors in Figure 7a for a selection of the best adsorbents.

As the samples showing good Cd^2+^ ions retention are those rich in Zn (EDX surface mapping results [29] are recalculated to 1.15 at.% Zn for cMOF.EBa, to 4.0 at.% Zn for cMOF.EBb, and to 8.6 at.% Zn for cMOF.EBc), the exchange of Zn^2+^ and Cd^2+^ ions is most likely a crucial process in the Cd^2+^ adsorption mechanism [54,55,56]. The Cd^2+^ ion is heavier and larger than the Zn^2+^ ion, but their chemical similarity allows Cd^2+^ to substitute for Zn^2+^ relatively easily [54], and it has been observed for Cd^2+^ adsorption on waste materials [55]. Additionally, Zn may be used as toxicity protection for Cd poisoning in animal studies [57], extending the environmental applicability of tested C-(MOF-5/PANI) samples.

Considering that the best Cd^2+^ adsorption was observed in the most acidic solution at pH 3, where the solubility of ZnO is the highest, a part of ZnO in the composite can be dissolved at pH 3 converting into Zn^2+^ ions in solution [58]. The gradual formation of Zn^2+^ enables the exchange with Cd^2+^ ions in the carbon scaffold. The mechanism consists of ZnO particles dissolution via hydrolysis to Zn^2+^ ions with Cd^2+^ ions taking their place, having in mind their almost identical hydration sphere size [59]. 

An interesting change in pH value compared to the initially set value of pH = 3 (for Cd^2+^ stock solution) is observed during the adsorption process. Namely, efficiency increases with ΔpH, and only samples that go through a substantial change in pH, ranging from 3–4 pH units, are highly efficient for Cd^2+^ removal (Figure 7a). Also, we observed that the adsorption capacity rises with increasing pre-carbonization MOF-5 content in composite precursors (Figure 7a) and can be associated with the proton-exchange process accompanying Cd^2+^ ions retention. The probable route may be denoted as the ‘Zn^2+^-Cd^2+^ exchange effect’, since the acid-leached C-(MOF-5/PANI) sample, where Zn was almost completely removed from the carbonaceous part of the composite [29], showed no adsorption (results not shown). 

To test this hypothesis, the Zn content in the post-adsorption supernatants was monitored, Figure 7b. In the samples with the highest Cd^2+^ retention (cMOF.EBc and cMOF), Zn^2+^ concentration in the supernatants after adsorption was the lowest measured (2.6–2.9 mg/L). These samples have high zinc (i.e., ZnO) content and a large specific surface area and pore volumes, while subtle differences in porosity and structure [29] direct the mechanism of retention. It can be envisaged that some of the pores cannot be penetrated by Cd^2+^ ions; therefore, H^+^ ions preserve the charge balance entering the pores with a concomitant rise in the pH of the supernatant. With rising pH, however, comes the formation of Zn-oxyhydroxide complexes that get dissolved Zn^2+^ to redeposit on the surface [53], which explains the lowest Zn^2+^ concentration in supernatants for the cMOF.EBc and cMOF samples, Figure 7b. Lesser Cd^2+^ removal, measured for cMOF.EBb and cMOF.EBa, stems from a lower amount of zinc, larger pore diameter, and lower S_BET_, which in turn induces a small rise in pH. Zinc ions remain in the solution as redeposition cannot take place due to solution acidity, and we measure higher zinc ion concentrations in the supernatant. 

Another explanation for the acidity changes accompanying Cd^2+^ removal may be related to the content of the oxygen- and nitrogen-containing groups in the cMOF.ESb cMOF.EB series and cMOF and their availability for protonation. The oxygen present in carbonyl and carboxyl groups may also play a role. We see that oxygen content is the lowest for the cMOF.ESb sample, with also the lowest S_BET_ and pore volume, which in turn has the slightest pH change of supernatant. 

MOF structures are found to be efficient for explored applications; for instance, Cu-MOF performance in Cd^2+^ removal showed efficiency of over 98%, and 2 mg/g capacity. However, this significant adsorption performance has been accomplished with a substantial adsorbent dosage of 0.5 g, significantly higher than the amount of 5 mg applied here. 

### 3.7. Materials Efficiency as Adsorbents in Pollutant Mixtures 

To discriminate whether the role of different adsorption centers may suit the ability of tested materials for complex mixture remediations, we have tested the adsorption capacity of C-(MOF-5/PANI) composites in a mixture of examined pollutants. The acidity was not adjusted since buffering introduces a new set of ions that can competitively bind to adsorbent active sites. Natural and anthropogenic pollutant occurrence targets low concentrations, and the 10 mg/L loading was used for all three contaminants. We tested a selection of the best-performing adsorbents for pollutant removal. Pesticide AA in a mixture with Cd, MB, or both contaminants is adsorbed at no less than 99% except for slightly lower results for cMOF.EBa (93%) in the AA/Cd mixture. Similarly, a nearly complete removal of MB is observed in two and three-component mixtures for all samples. It can be gleaned that the high surface area of the materials tested here is suitable for the adsorption of contaminants in environmental occurrence doses.

**Figure 8:**
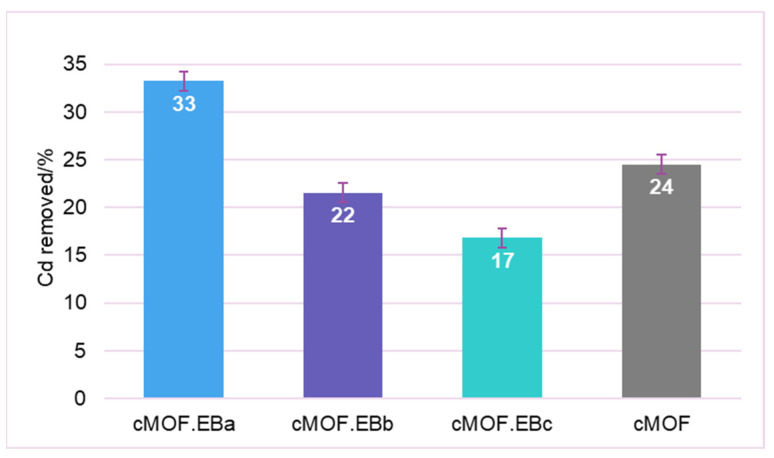
Results for Cd^2+^ ions removal in AA/Cd/MB mixtures by C-(MOF-5/PANI) and cMOF samples.

Visible differences in the adsorption performance in mixtures are seen in Cd^2+^ ions removal. The incomplete adsorption of Cd^2+^ is firstly recorded in the AA/Cd pollutant mixture where cMOF.EBa and cMOF removed 67% and 58%, respectively, which can be directly related to their values of S_BET_. Both samples showed higher removal than cMOF.EBb (19.7%) and cMOF.EBc (22.5%). The results regarding Cd^2+^ ions removal in the AA/Cd/MB mixture are given in Figure 8. The highest amount of adsorbed Cd^2+^ is recorded for cMOF.EBa, 33%, while somewhat lower values are seen for cMOF, cMOF.EBb, and cMOF.EBc. An additional lowering of the adsorption capacity in the AA/Cd/MB mixture, in comparison to AA/Cd, is observed, and it is reasonable to assume that AA and MB deposition at the surface of nanomaterials by hydrogen bonding hinders Cd^2+^/Zn^2+^ exchange and reduces Cd^2+^ removal. Thus, in tested materials, pollutants in their mixture competitively bind to adsorption sites that favor AA and MB over metal cations. It is interesting that for the pollutant mixtures, enhanced adsorption of Cd^2+^ was found for the composite, cMOF.EBa, compared to cMOF.

These results highlight the versatility of applications of functional nanocarbons prepared by carbonization of PANI/MOF composite materials. Complete removal of pesticide and dye at low 10 mg/L concentrations is shown in the presence of cadmium metal ions. On the other hand, metal ions are sensitive to suspension composition and other pollutants’ presence; thus, their removal is a specific challenge in complex contaminated environments.

To the best of our knowledge, there is only one report dealing with composites of magnetic chitosan/nanocarbons/UiO-66 MOFs that were utilized for the simultaneous removal of cobalt, malachite green, and imidacloprid. Optimized experimental results have led to adsorption capacities between 25 and 62 mg/g [60]. Although different types of pollutants may be tested in a single study, they are often addressed separately [61]. The lack of this type of study in the literature is associated with complex pollutant systems and may be perceived as a new challenge set before scientists working in material design for environmental applications.

## 4. Conclusions

The environmental application of carbonized MOF-5/PANI composites, C-(MOF-5/PANI), is tested. Composites were prepared from emeraldine salt (ES) or the base (EB) form of conductive polymer PANI, which are N,O-doped carbon/ZnO/ZnS and N,O-doped carbon/ZnO composites, respectively. Prepared materials have a mean particle size in the 112–145 nm range, high surface area, and pore volume with dominant mesoporosity. Functional carbonized composites were employed for the adsorption of pesticide, dye, and metal cation in the batch adsorption study. The results for acetamiprid pesticide (AA) adsorption showed that the cMOF.EB composites exhibited excellent adsorption (over 100 mg/g). The best result at high pesticide concentrations was obtained for the cMOF sample (140 mg/g), while the composite cMOF.EBa showed matching results at environmentally significant AA loadings. Moreover, the estimated isotherm monolayer capacity of cMOF.EBa composite is twice the value calculated for cMOF. 

Subsequently, materials were tested for the removal of Methylene Blue (MB) dye. As the MOF-5 content increased in the composite precursors of the cMOF.EB composites, the adsorption of MB decreased, from 68 to 36 wt.%, while the cMOF sample achieved 74 wt.% MB removal. This trend is similar to the findings for AA retention and is associated with the development of the surface area and porosity. The presence of oxygen/nitrogen groups on the surface plays a crucial role in nitrogen-rich AA and MB adsorption via hydrogen bonding.

A somewhat different trend is observed for Cd^2+^ ions removal, which increased with the MOF-5 content in the precursors synthesized from EB, up to 98% removal for composite produced with high MOF-5 content. The proton-exchange process, accompanying Cd^2+^ retention, associated with pH changes is in direct relation to the presence of ZnO in the sample and its partial conversion into Zn^2+^ ions in the solution. The results provided insights into the mechanisms involved—surface area/porosity and hydrogen bonding are crucial for pesticide and dye adsorption, while proton and Zn^2+^/Cd^2+^ ion exchange, in addition to oxygen/nitrogen-containing groups, were responsible for Cd^2+^ retention.

In the mixtures of pollutants, pesticide, and dye, molecules are almost completely removed by the tested carbonized composites, while incomplete retention of Cd^2+^ is observed. The cMOF.EBa sample accomplished the highest Cd^2+^ removal efficiency (33%) due to its high surface area and pore volumes. Competitive binding between the pollutants on the adsorption sites favors pesticide and dye molecules and disables the ion exchange necessary for metal cations removal.

This study demonstrated the potential of composite materials derived from MOF/PANI composites where N-doping, enabled through the PANI component, is important for the efficient removal of emerging environmental contaminants. The findings highlighted the importance of surface area, porosity, composition, and functional groups for the accomplished adsorption capacity of the materials. Thus, these carbon-based composite adsorbents hold promise for the active removal of pollutants from aqueous environments. 

## Figures and Tables

**Table 1:** Textural properties of C-(MOF-5/PANI) composites and cMOF.

	cMOF.ESb	cMOF.EBa	cMOF.EBb	cMOF.EBc	cMOF
Total pore volume (Gurvich at 0.98 p/p^0^) V_tot_ (cm^3^/g)	0.315	1.106	0.464	0.359	0.715
Specific surface area (B.E.T. method) S_BET_ (m^2^/g)	393	611	471	413	550
Mesopore volume (B.J.H. Desorption) V_meso_ (cm^3^/g)	0.169	1.047	0.259	0.201	0.584
Median mesopore diameter (B.J.H. Desorption) D_meso_ (nm)	5.8	10.7	12.3	12.5	9.4
Micropore volume (Horvath and Kawazoe) V_mic_ (cm^3^/g)	0.164	0.251	0.194	0.170	0.225

## Data Availability

The data presented in this study are available on request from the corresponding author.

## Supplementary Materials

The following supporting information can be downloaded at: https://www.mdpi.com/article/10.3390/polym15224349/s1, Figure S1: N_2_ adsorption/desorption isotherms for C-(MOF-5/PANI) composites and cMOF; Figure S2: BJH curves for pore size distribution (desorption branch of isotherm) with derivative profiles, given in red, for C-(MOF-5/PANI) composites and cMOF; Figure S3: Percent of Cd^2+^ ions removed after adsorption on selected samples, measured for different suspension acidities, pH = 3, 5, and 8.

## References

[1] Milojević-Rakić M., Bajuk-Bogdanović D. (2023). Recent Advances in Zeolites and Porous Materials Applications in Catalysis and Adsorption Processes. Catalysts.

[2] Milojević-Rakić M., Popadić D., Janošević Ležaić A., Jevremović A., Nedić Vasiljević B., Uskoković-Marković S., Bajuk-Bogdanović D. (2022). MFI, BEA and FAU Zeolite Scavenging Role in Neonicotinoids and Radical Species Elimination. Environ. Sci. Process Impacts.

[3] Jevremović A., Stanojković A., Arsenijević D., Arsenijević A., Arzumanyan G., Mamatkulov K., Petrović J., Nedić Vasiljević B., Bajuk-Bogdanović D., Milojević-Rakić M. (2022). Mitigating Toxicity of Acetamiprid Removal Techniques—Fe Modified Zeolites in Focus. J. Hazard. Mater..

[4] Popadić D., Gavrilov N., Krstić J., Nedić Vasiljević B., Janošević Ležaić A., Uskoković-Marković S., Milojević-Rakić M., Bajuk-Bogdanović D. (2023). Spectral Evidence of Acetamiprid’s Thermal Degradation Products and Mechanism. Spectrochim. Acta Part A Mol. Biomol. Spectrosc..

[5] Naghdi S., Shahrestani M.M., Zendehbad M., Djahaniani H., Kazemian H., Eder D. (2023). Recent Advances in Application of Metal-Organic Frameworks (MOFs) as Adsorbent and Catalyst in Removal of Persistent Organic Pollutants (POPs). J. Hazard. Mater..

[6] Yusuf V.F., Malek N.I., Kailasa S.K. (2022). Review on Metal–Organic Framework Classification, Synthetic Approaches, and Influencing Factors: Applications in Energy, Drug Delivery, and Wastewater Treatment. ACS Omega.

[7] Au V.K.-M. (2020). Recent Advances in the Use of Metal-Organic Frameworks for Dye Adsorption. Front. Chem..

[8] Beydaghdari M., Hooriabad Saboor F., Babapoor A., Karve V., Asgari M. (2022). Recent Advances in MOF-Based Adsorbents for Dye Removal from the Aquatic Environment. Energies.

[9] Mansoorianfar M., Nabipour H., Pahlevani F., Zhao Y., Hussain Z., Hojjati-Najafabadi A., Hoang H.Y., Pei R. (2022). Recent Progress on Adsorption of Cadmium Ions from Water Systems Using Metal-Organic Frameworks (MOFs) as an Efficient Class of Porous Materials. Environ. Res..

[10] Alshorifi F.T., El Dafrawy S.M., Ahmed A.I. (2022). Fe/Co-MOF Nanocatalysts: Greener Chemistry Approach for the Removal of Toxic Metals and Catalytic Applications. ACS Omega.

[11] Mohan B., Kamboj A., Virender, Singh K., Priyanka, Singh G., Pombeiro A.J.L., Ren P. (2023). Metal-Organic Frameworks (MOFs) Materials for Pesticides, Heavy Metals, and Drugs Removal: Environmental Safety. Sep. Purif. Technol..

[12] Pauletto P.S., Bandosz T.J. (2022). Activated Carbon versus Metal-Organic Frameworks: A Review of Their PFAS Adsorption Performance. J. Hazard. Mater..

[13] Nabipour H., Rohani S., Batool S., Yusuff A.S. (2023). An Overview of the Use of Water-Stable Metal-Organic Frameworks in the Removal of Cadmium Ion. J. Environ. Chem. Eng..

[14] Fang Q.-R., Yuan D.-Q., Sculley J., Li J.-R., Han Z.-B., Zhou H.-C. (2010). Functional Mesoporous Metal−Organic Frameworks for the Capture of Heavy Metal Ions and Size-Selective Catalysis. Inorg. Chem..

[15] Wang K., Gu J., Yin N. (2017). Efficient Removal of Pb(II) and Cd(II) Using NH 2 -Functionalized Zr-MOFs via Rapid Microwave-Promoted Synthesis. Ind. Eng. Chem. Res..

[16] Salimi M., Behbahani M., Sobhi H.R., Ghambarian M., Esrafili A. (2020). Trace Measurement of Lead and Cadmium Ions in Wastewater Samples Using a Novel Dithizone Immobilized Metal–Organic Framework-based Μ-dispersive Solid-phase Extraction. Appl. Organomet. Chem..

[17] Sánchez-Sánchez Á., Suárez-García F., Martínez-Alonso A., Tascón J.M.D. (2015). Synthesis, Characterization and Dye Removal Capacities of N-Doped Mesoporous Carbons. J. Colloid Interface Sci..

[18] dos Reis G.S., Bergna D., Grimm A., Lima E.C., Hu T., Naushad M., Lassi U. (2023). Preparation of Highly Porous Nitrogen-Doped Biochar Derived from Birch Tree Wastes with Superior Dye Removal Performance. Colloids Surfaces A Physicochem. Eng. Asp..

[19] Fujisaki T., Kashima K., Serrano-Luginbühl S., Kissner R., Bajuk-Bogdanović D., Milojević-Rakić M., Ćirić-Marjanović G., Busato S., Lizundia E., Walde P. (2019). Effect of Template Type on the Preparation of the Emeraldine Salt Form of Polyaniline (PANI-ES) with Horseradish Peroxidase Isoenzyme C (HRPC) and Hydrogen Peroxide. RSC Adv..

[20] Ćirić-Marjanović G., Milojević-Rakić M., Janošević-Ležaić A., Luginbühl S., Walde P. (2017). Enzymatic Oligomerization and Polymerization of Arylamines: State of the Art and Perspectives. Chem. Pap..

[21] Pašti I., Milojević-Rakić M., Junker K., Bajuk-Bogdanović D., Walde P., Ćirić-Marjanović G. (2017). Superior Capacitive Properties of Polyaniline Produced by a One-Pot Peroxidase/H_2_O_2_-Triggered Polymerization of Aniline in the Presence of AOT Vesicles. Electrochim. Acta.

[22] Radoičić M., Ćirić-Marjanović G., Spasojević V., Ahrenkiel P., Mitrić M., Novaković T., Šaponjić Z. (2017). Superior Photocatalytic Properties of Carbonized PANI/TiO_2_ Nanocomposites. Appl. Catal. B Environ..

[23] Jović A., Milikić J., Bajuk-Bogdanović D., Milojević-Rakić M., Nedić Vasiljević B., Krstić J., Cvjetićanin N., Šljukić B. (2018). 12-Phosphotungstic Acid Supported on BEA Zeolite Composite with Carbonized Polyaniline for Electroanalytical Sensing of Phenols in Environmental Samples. J. Electrochem. Soc..

[24] Jevremović A., Bober P., Mičušík M., Kuliček J., Acharya U., Pfleger J., Milojević-Rakić M., Krajišnik D., Trchová M., Stejskal J. (2019). Synthesis and Characterization of Polyaniline/BEA Zeolite Composites and Their Application in Nicosulfuron Adsorption. Microporous Mesoporous Mater..

[25] Milojević-Rakić M., Janošević A., Krstić J., Nedić Vasiljević B., Dondur V., Ćirić-Marjanović G. (2013). Polyaniline and Its Composites with Zeolite ZSM-5 for Efficient Removal of Glyphosate from Aqueous Solution. Microporous Mesoporous Mater..

[26] Shanahan J., Kissel D.S., Sullivan E. (2020). PANI@UiO-66 and PANI@UiO-66-NH 2 Polymer-MOF Hybrid Composites as Tunable Semiconducting Materials. ACS Omega.

[27] Ramohlola K.E., Monana G.R., Hato M.J., Modibane K.D., Molapo K.M., Masikini M., Mduli S.B., Iwuoha E.I. (2018). Polyaniline-Metal Organic Framework Nanocomposite as an Efficient Electrocatalyst for Hydrogen Evolution Reaction. Compos. Part B Eng..

[28] Biserčić M.S., Marjanović B., Zasońska B.A., Stojadinović S., Ćirić-Marjanović G. (2020). Novel Microporous Composites of MOF-5 and Polyaniline with High Specific Surface Area. Synth. Met..

[29] Savić M., Janošević Ležaić A., Gavrilov N., Pašti I., Nedić Vasiljević B., Krstić J., Ćirić-Marjanović G. (2023). Carbonization of MOF-5/Polyaniline Composites to N,O-Doped Carbon/ZnO/ZnS and N,O-Doped Carbon/ZnO Composites with High Specific Capacitance, Specific Surface Area and Electrical Conductivity. Materials.

[30] Wang J., Wang Y., Hu H., Yang Q., Cai J. (2020). From Metal–Organic Frameworks to Porous Carbon Materials: Recent Progress and Prospects from Energy and Environmental Perspectives. Nanoscale.

[31] Trukawka M., Cendrowski K., Peruzynska M., Augustyniak A., Nawrotek P., Drozdzik M., Mijowska E. (2019). Carbonized Metal–Organic Frameworks with Trapped Cobalt Nanoparticles as Biocompatible and Efficient Azo-Dye Adsorbent. Environ. Sci. Eur..

[32] Biserčić M.S., Marjanović B., Vasiljević B.N., Mentus S., Zasońska B.A., Ćirić-Marjanović G. (2019). The Quest for Optimal Water Quantity in the Synthesis of Metal-Organic Framework MOF-5. Microporous Mesoporous Mater..

[33] Lecloux A., Pirard J.P. (1979). The Importance of Standard Isotherms in the Analysis of Adsorption Isotherms for Determining the Porous Texture of Solids. J. Colloid Interface Sci..

[34] Horváth G., Kawazoe K. (1983). Method for the Calculation of Effective Pore Size Distribution in Molecular Sieve Carbon. J. Chem. Eng. Jpn..

[35] Drozdov V.A., Fenelonov V.B., Okkel L.G., Gulyaeva T.I., Antonicheva N.V., Sludkina N.S. (1998). Investigation of Reference Catalysts in Boreskov Institute of Catalysis: Texture of Reference Platinum Catalysts. Appl. Catal. A Gen..

[36] Kubier A., Wilkin R.T., Pichler T. (2019). Cadmium in Soils and Groundwater: A Review. Appl. Geochem..

[37] Filipe V., Hawe A., Jiskoot W. (2010). Critical Evaluation of Nanoparticle Tracking Analysis (NTA) by NanoSight for the Measurement of Nanoparticles and Protein Aggregates. Pharm. Res..

[38] Malloy A. (2011). Count, Size and Visualize Nanoparticles. Mater. Today.

[39] Gong Y.-T., Li B.-H., Pei T., Lin C.-H., Lee S. (2016). Raman Investigation on Carbonization Process of Metal-Organic Frameworks. J. Raman Spectrosc..

[40] Ferrari A.C., Robertson J. (2000). Interpretation of Raman Spectra of Disordered and Amorphous Carbon. Phys. Rev. B.

[41] Gavrilov N., Pašti I.A., Mitrić M., Travas-Sejdić J., Ćirić-Marjanović G., Mentus S.V. (2012). Electrocatalysis of Oxygen Reduction Reaction on Polyaniline-Derived Nitrogen-Doped Carbon Nanoparticle Surfaces in Alkaline Media. J. Power Sources.

[42] Cuscó R., Alarcón-Lladó E., Ibáñez J., Artús L., Jiménez J., Wang B., Callahan M.J. (2007). Temperature Dependence of Raman Scattering in ZnO. Phys. Rev. B.

[43] Najib S., Bakan F., Abdullayeva N., Bahariqushchi R., Kasap S., Franzò G., Sankir M., Demirci Sankir N., Mirabella S., Erdem E. (2020). Tailoring Morphology to Control Defect Structures in ZnO Electrodes for High-Performance Supercapacitor Devices. Nanoscale.

[44] Dolatabadi M., Naidu H., Ahmadzadeh S. (2021). A Green Approach to Remove Acetamiprid Insecticide Using Pistachio Shell-Based Modified Activated Carbon; Economical Groundwater Treatment. J. Clean. Prod..

[45] Mohammad S.G., Ahmed S.M., Amr A.E.-G.E., Kamel A.H. (2020). Porous Activated Carbon from Lignocellulosic Agricultural Waste for the Removal of Acetampirid Pesticide from Aqueous Solutions. Molecules.

[46] Choumane F.Z., Benguella B. (2014). Removal of Acetamiprid from Aqueous Solutions with Low-Cost Sorbents. Desalin. Water Treat..

[47] Popadić D., Gavrilov N., Ignjatović L., Krajišnik D., Mentus S., Milojević-Rakić M., Bajuk-Bogdanović D. (2022). How to Obtain Maximum Environmental Applicability from Natural Silicates. Catalysts.

[48] Li Y., Gao C., Jiao J., Cui J., Li Z., Song Q. (2021). Selective Adsorption of Metal–Organic Framework toward Methylene Blue: Behavior and Mechanism. ACS Omega.

[49] Haque E., Jun J.W., Jhung S.H. (2011). Adsorptive Removal of Methyl Orange and Methylene Blue from Aqueous Solution with a Metal-Organic Framework Material, Iron Terephthalate (MOF-235). J. Hazard. Mater..

[50] EL-Mekkawi D.M., Ibrahim F.A., Selim M.M. (2016). Removal of Methylene Blue from Water Using Zeolites Prepared from Egyptian Kaolins Collected from Different Sources. J. Environ. Chem. Eng..

[51] Ullah A., Zahoor M., Din W.U., Muhammad M., Khan F.A., Sohail A., Ullah R., Ali E.A., Murthy H.C.A. (2022). Removal of Methylene Blue from Aqueous Solution Using Black Tea Wastes: Used as Efficient Adsorbent. Adsorpt. Sci. Technol..

[52] Zhu Y., Yi B., Yuan Q., Wu Y., Wang M., Yan S. (2018). Removal of Methylene Blue from Aqueous Solution by Cattle Manure-Derived Low Temperature Biochar. RSC Adv..

[53] Sheela T., Nayaka Y.A., Viswanatha R., Basavanna S., Venkatesha T.G. (2012). Kinetics and Thermodynamics Studies on the Adsorption of Zn(II), Cd(II) and Hg(II) from Aqueous Solution Using Zinc Oxide Nanoparticles. Powder Technol..

[54] Tang L., Qiu R., Tang Y., Wang S. (2014). Cadmium–Zinc Exchange and Their Binary Relationship in the Structure of Zn-Related Proteins: A Mini Review. Metallomics.

[55] Atar N., Olgun A., Wang S. (2012). Adsorption of Cadmium (II) and Zinc (II) on Boron Enrichment Process Waste in Aqueous Solutions: Batch and Fixed-Bed System Studies. Chem. Eng. J..

[56] Mohan D., Singh K.P. (2002). Single- and Multi-Component Adsorption of Cadmium and Zinc Using Activated Carbon Derived from Bagasse—An Agricultural Waste. Water Res..

[57] Kudo N., Yamashina S., Waku K. (1986). Protection against Cadmium Toxicity by Zinc: Decrease in the Cd-High Molecular Weight Protein Fraction in Rat Liver and Kidney on Zn Pretreatment. Toxicology.

[58] Cardoso D., Narcy A., Durosoy S., Bordes C., Chevalier Y. (2021). Dissolution Kinetics of Zinc Oxide and Its Relationship with Physicochemical Characteristics. Powder Technol..

[59] De Carvalho Izidoro J., Fungaro D.A., Wang S. (2011). Bin Zeolite Synthesis from Brazilian Coal Fly Ash for Removal of Zn^2+^ and Cd^2+^ from Water. Adv. Mater. Res..

[60] Motaghi H., Arabkhani P., Parvinnia M., Asfaram A. (2022). Simultaneous Adsorption of Cobalt Ions, Azo Dye, and Imidacloprid Pesticide on the Magnetic Chitosan/Activated Carbon@UiO-66 Bio-Nanocomposite: Optimization, Mechanisms, Regeneration, and Application. Sep. Purif. Technol..

[61] Abdelhameed R.M., Emam H.E. (2022). Modulation of Metal Organic Framework Hybrid Cotton for Efficient Sweeping of Dyes and Pesticides from Wastewater. Sustain. Mater. Technol..

