# Supplementary material for: Environmental Potential of Carbonized MOF-5/PANI Composites for Pesticide, Dye, and Metal Cations—Can They Actually Retain Them All?
Source: Polymers (Basel). 2023 Nov 7;15(22):4349. doi: 10.3390/polym15224349 (PMC10675784; doi:10.3390/polym15224349)
Supplement: Supplementary file 1 [file polymers-15-04349-s001.zip › polymers-2677035-supplementary.pdf]

polymers

MDPI

Supplementary material

Environmental Potential of Carbonized MOF-5/PANI Composites for Pesticide, Dye, and Metal Cations—Can They Actually Retain Them All?

Anka Jevremović [1], Marjetka Savić [2], Aleksandra Janošević Ležaić [3], Jugoslav Krstić [4], Nemanja Gavrilov [1], Danica Bajuk-Bogdanović [1], Maja Milojević-Rakić [1,]* and Gordana Ćirić-Marjanović [1]

[1] University of Belgrade-Faculty of Physical Chemistry, Studentski trg 12-16, 11158 Belgrade, Serbia;
[2] Vinča Institute of Nuclear Science, University of Belgrade, National Institute of the Republic of Serbia, P.O. Box 522, 11001 Belgrade, Serbia;
[3] University of Belgrade-Faculty of Pharmacy, Vojvode Stepe 450, 11221 Belgrade, Serbia;
[4] University of Belgrade, Institute of Chemistry, Technology and Metallurgy, Department of Catalysis and Chemical Engineering, Njegoševa 12, 11000 Belgrade, Serbia;
* Correspondence: maja@@ffh.bg.ac.rs

Citation: Jevremović, A.; Savić, M.; Janošević Ležaić, A.; Krstić, J.; Gavrilov, N.; Bajuk-Bogdanović, D.; Milojević-Rakić, M.; Ćirić-Marjanović, G. Environmental Potential of Carbonized MOF-5/PANI Composites for Pesticide, Dye, and Metal Cations—Can They Actually Retain Them All? *Polymers* **2023**, *15*, x. https://doi.org/10.3390/xxxxx

Academic Editors: Qina Sun, Lichun Xiao and Liazhou Song

Received: 7 October 2023
Revised: 31 October 2023
Accepted: 1 November 2023
Published: 3 November 2023

cc BY

Copyright: © 2023 by the authors. Licensee MDPI, Basel, Switzerland. This article is an open access article distributed under the terms and conditions of the Creative Commons Attribution (CC BY) license (https://creativecommons.org/licenses/by/4.0/).

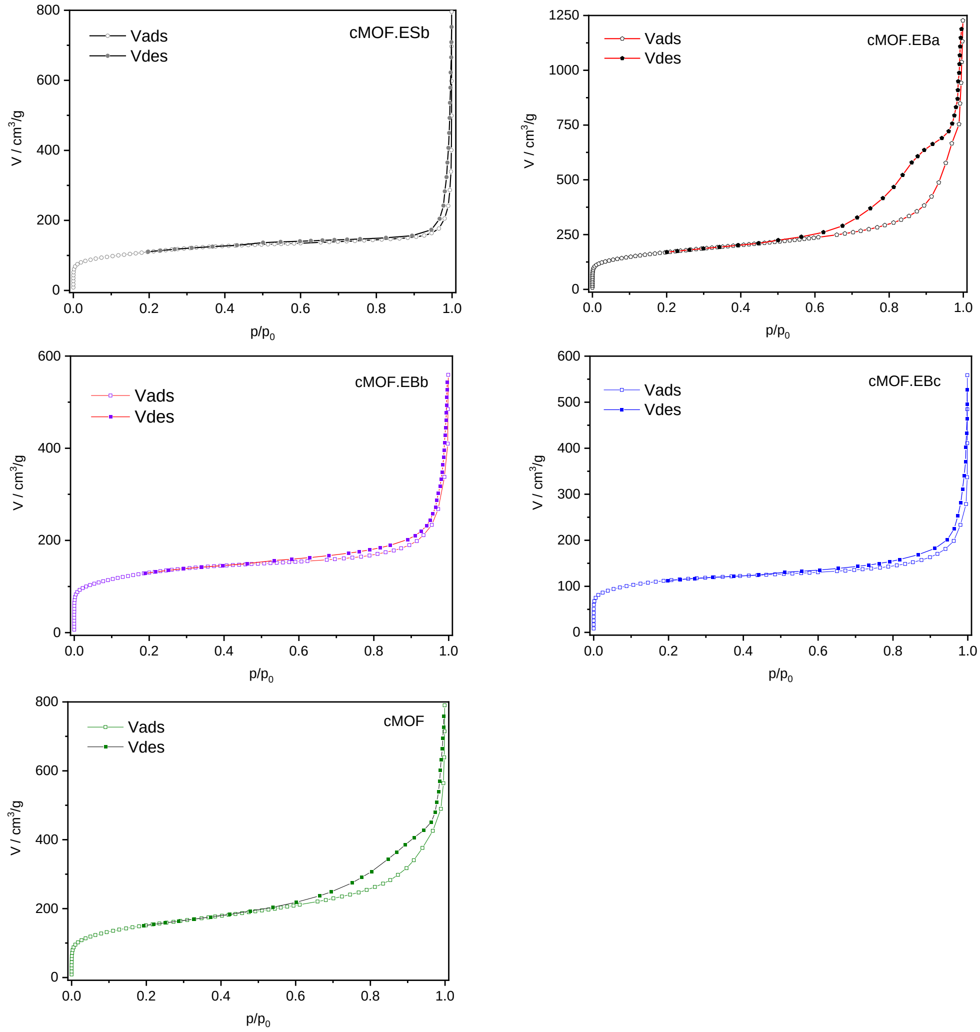

Figure S1. N_2 adsorption/desorption isotherms for C-(MOF-5/PANI) composites and cMOF.

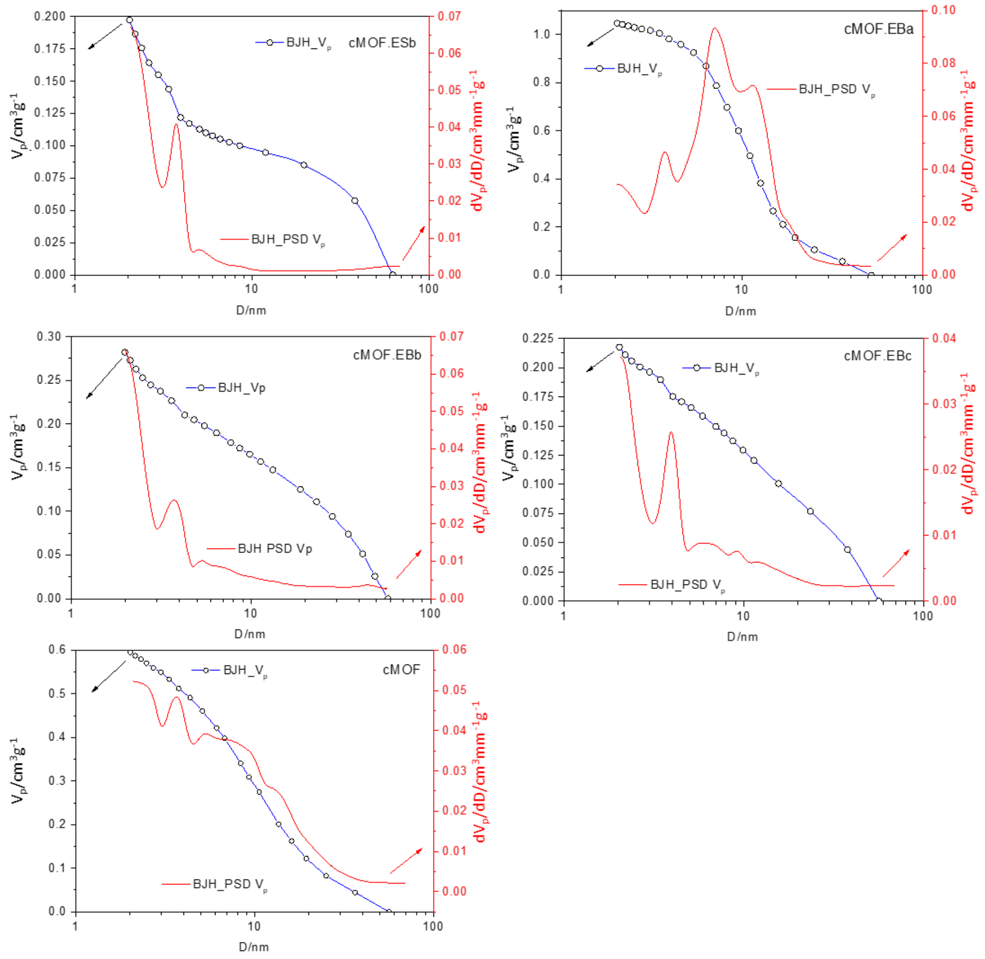

Figure S2. B. J. H. curves for pore size distribution (desorption branch of isotherm) with derivative profiles, given in red, for C-(MOF-5/PANI) composites and cMOF.

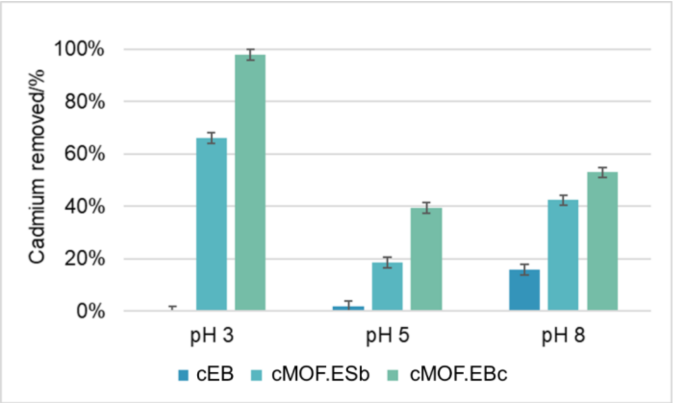

Figure S3. Percent of Cd^{2+} ions removed after adsorption on selected samples, measured for different suspension acidities, pH = 3, 5 and 8.
